# Ex Vivo Expansion of Functional Hematopoietic Stem Cells, Facilitating Transplantation in the Absence of Conditioning

**DOI:** 10.1097/HS9.0000000000000306

**Published:** 2019-10-10

**Authors:** Michael D. Milsom

**Affiliations:** 1 Division of Experimental Hematology, German Cancer Research Center (DKFZ), Heidelberg, Germany; 2 Heidelberg Institute for Stem Cell Technology and Experimental Medicine (HI-STEM), Heidelberg, Germany.

The in vitro expansion of functional hematopoietic stem cells (HSCs) can be thought of as a holy grail of the experimental hematology research community, since it would potentially facilitate transplantation from donor sources where the number of HSCs can be limiting, such as umbilical cord blood. Indeed, much effort has been expended in trying to devise novel strategies to enhance the culture of primary HSCs, while maintaining their multipotent engraftment capacity. The use of various cytokine and growth factor combinations, small molecule agonists and antagonists, co-culture with stromal feeder cells and modulation of oxygen tension, are amongst the range of strategies that have been employed to enhance the growth of HSCs in vitro. However, the identification of a robust strategy to achieve these ends, that is, both GMP compliant and can be scaled towards clinical application, remains elusive. In a recent letter in the journal Nature, Wilkinson and colleagues describe a new approach that is based upon slight modifications to existing HSC in vitro culture methodologies, in concert with the novel use of the soluble synthetic polymer, polyvinyl alcohol (PVA), as a replacement for serum albumin.^1^ They report that this technique facilitates the several hundred-fold expansion of functional murine HSCs in vitro, for culture periods that span several weeks. Evidence is presented to suggest that this approach may also work for human HSCs and the methodology is relatively straightforward, using defined reagents that should be amenable to clinical translation.

In order to systematically modify existing HSC culture conditions, the authors first carried out a simple titration experiment in serum free medium using recombinant murine stem cell factor (SCF) and thrombopoietin (TPO), which have previously been used as a minimal cytokine cocktail to maintain HSCs in vitro. In serum free conditions, it was found that while high concentrations of TPO were favorable for HSC expansion, more limiting levels of SCF were optimal. This surprising finding was not in line with the conventional wisdom that such cytokines should be used within the range of concentrations where stimulatory effects have plateaued out, with the authors speculating that lower levels of SCF did not promote internalization of its receptor, c-Kit, thus facilitating sustained signal transduction.

Next, the authors assessed whether it was better to completely replace the culture medium every few days, rather than only replacing half of the culture media. In the latter approach, the older media that is carried forward in culture is considered to have been “conditioned”, in that soluble factors secreted by the cells are thought to further promote HSC growth. However, the experimental data again confounded expectations, in that HSCs subject to complete medium changes performed better than those in which conditioned media was retained. In line with this finding, it was found that pro-inflammatory cytokines, such as IL-1β and IL-6, accumulated in the culture media and could be a driver of HSC loss, for example by promoting myeloid differentiation. In order to facilitate these regular changes of culture media, which would wash out these inhibitory cytokines, the authors additionally coated tissue culture wells with fibronectin to enhance HSC adhesion.

Finally, the authors sought to replace the recombinant human serum albumin (HAS) that is used as “carrier molecule” in culture medium, but which the authors hypothesized was also a source of contaminants that might promote the production of inhibitory pro-inflammatory cytokines. After screening a range of chemically synthesized candidate molecules, they found that supplementing culture media with PVA significantly enhanced the performance of expanded HSCs, compared to cells that had been grown in cultures that instead contained HAS. This correlated with lower levels of secretion of pro-inflammatory cytokines.

Having incorporated all of these changes, limiting dilution transplantation experiments were then performed to quantify the precise degree of HSC expansion. Over a 28-day culture period, it was found that immunophenotypic HSCs expanded by a factor of approximately 8000-fold, while the transplantation experiments revealed that functional HSCs, capable of long-term multi-lineage engraftment, had expanded by a factor of between 236- and 899-fold compared to input levels. The authors then conducted secondary transplantations in order to add a further level of stringency onto the characterization of HSC functionality and could demonstrate robust engraftment, corresponding to an estimated in vitro expansion of serially transplantable HSCs in the range of 56- and 204-fold compared to freshly isolated input cells. Taken together, these results indicate that this protocol for in vitro culture of murine HSCs leads to an unprecedented expansion of functionally potent cells. Additional experiments suggested that human cord blood-derived HSCs could also be propagated more efficiently in vitro using this methodology when compared to HAS-containing culture medium, as assessed by engraftment levels following transplantation into immune-deficient mice. Finally, the authors assessed engraftment into non-conditioned recipients using either 50 freshly isolated murine HSCs, or the total progeny that 50 HSCs generate after 28 days of in vitro expansion using the newly devised protocol described above. While the freshly isolated HSCs failed to contribute appreciable levels of engraftment in this transplantation setting, the expanded HSCs were able to compete with the endogenous hematopoietic cells, resulting in between 5% and 15% donor chimerism in the peripheral blood at 16 weeks post-transplantation. This data suggests that the in vitro expanded HSCs may have similar functional potency to the endogenous HSCs.

This work is already generating significant excitement within the experimental hematology community, as it appears to represent a significant step forward from the current state of the art in terms of the function potency of the expanded cells that are produced using this protocol. The fact that this protocol is relatively simple, employing reagents that should be accessible to most laboratories, means that many groups will attempt to recapitulate these results and apply the methodology to a range of applications. It is certain that additional pre-clinical validation studies will follow and will be intriguing to follow the attempts to clinically translate these findings. Clearly this discovery has the potential to change the face of HSC transplantation, and may help the realization of cord blood banking for transplantation of adults. In addition, the capacity to maintain functional HSCs in culture could dramatically boost the application of ex-vivo gene editing, both for therapeutic and research purposes, while such culture conditions could also be used to capture and expand functional HSCs that have been generated by in vitro specification of pluripotent cell lines such as ES or iPS cells (Fig. 1).

**FIGURE 1:**
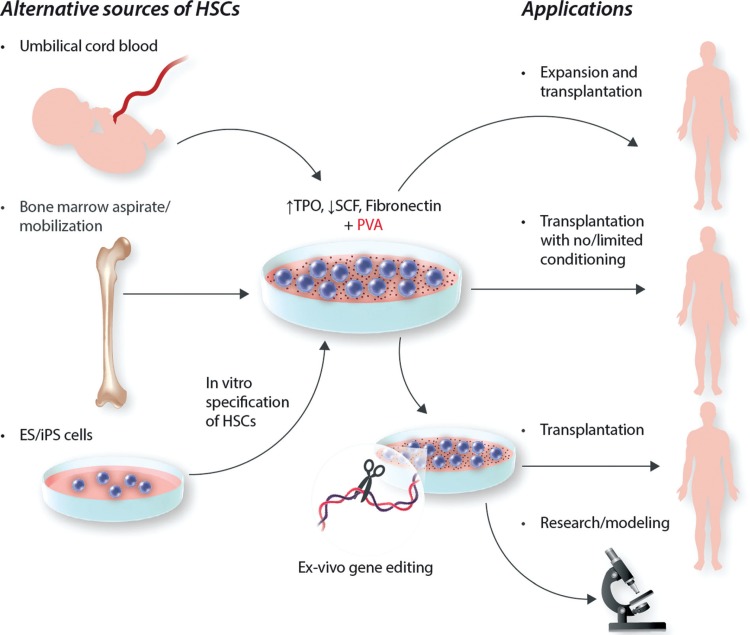
**Potential applications of in vitro HSC expansion and maintenance.** The capacity to expand functional HSCs in vitro may potentiate the use alternative sources of HSCs for transplantation, which have not been feasible in the past due to limiting numbers of cells with engraftment potential. Such alternative sources could include umbilical cord blood, or the setting of in vitro specification of HSCs from pluripotent cell lines such as embryonic stem (ES) cells or induced pluripotent stem (iPS) cells, which is currently quite inefficient. Alternatively, high input levels of donor HSCs may facilitate transplantation without the need for harsh conditioning regimens, which can ultimately drive both morbidity and mortality in patients. Finally, if functional HSCs can be faithfully maintained in vitro, then this could revolutionize the process of gene editing of HSCs for both clinical and research purposes, since such applications require extensive ex vivo manipulations which typically result in the loss of HSC potential.

## References

[1] Wilkinson AC Ishida R Kikuchi M Long-term ex vivo haematopoietic-stem-cell expansion allows nonconditioned transplantation. *Nature.* 2019;571:117–121. 31142833 10.1038/s41586-019-1244-x PMC7006049

